# More public health service providers are experiencing job burnout than clinical care providers in primary care facilities in China

**DOI:** 10.1186/s12960-020-00538-z

**Published:** 2020-12-03

**Authors:** Shan Lu, Liang Zhang, Niek Klazinga, Dionne Kringos

**Affiliations:** 1 School of Medicine and Health Management, Tongji Medical College, Huazhong University of Science and Technology, Wuhan, 430030 China; 2 Research Centre for Rural Health Service, Key Research Institute of Humanities and Social Sciences of Hubei Provincial Department of Education, Wuhan, 430030 China; 3 Department of Public and Occupational Health, Amsterdam UMC, Amsterdam Public Health Research Institute, University of Amsterdam, Amsterdam, The Netherlands

**Keywords:** Burnout, Primary care, Clinical care providers, Public health service providers

## Abstract

**Background:**

Health workers are at high risk of job burnout. Primary care in China has recently expanded its scope of services to a broader range of public health services in addition to clinical care. This study aims to measure the prevalence of burnout and identify its associated factors among clinical care and public health service providers at primary care facilities.

**Methods:**

A cross-sectional survey (2018) was conducted among 17,816 clinical care and public health service providers at 701 primary care facilities from six provinces. Burnout was measured by the Chinese version of the Maslach Burnout Inventory-General Scale, and multilevel linear regression analysis was conducted to identify burnout’s association with demographics, as well as occupational and organisational factors.

**Results:**

Overall, half of the providers (50.09%) suffered from burnout. Both the presence of burnout and the proportion of severe burnout among public health service providers (58.06% and 5.25%) were higher than among clinical care providers (47.55% and 2.26%, respectively). Similar factors were associated with burnout between clinical care and public health service providers. Younger, male, lower-educated providers and providers with intermediate professional title, permanent contract or higher working hours were related to a higher level of burnout. Organisational environment, such as the presence of a performance-based salary system, affected job burnout.

**Conclusions:**

Job burnout is prevalent among different types of primary care providers in China, indicating the need for actions that encompass the entirety of primary care. We recommend strengthening the synergy between clinical care and public health services and transforming the performance-based salary system into a more quality-based system that includes teamwork incentives.

## Background

Strengthening primary care (PC) to improve health system outcomes is high on the health policy agenda of most countries, and this is expected to be further reinforced in the aftermath of the current COVID-19 pandemic [1–3]. This is also true for China. The Chinese government launched a new round of healthcare reform in 2009, with priority given to the strengthening of PC. Two out of five major reform programmes were directly related to PC. One of the programmes involved activities to construct PC facilities and to strengthen its workforce, and the other one re-established the importance of public health services within PC via the issuance of an essential package of services. As a result of this policy, PC services provided by PC facilities in China can be classified into two distinct categories, namely, basic clinical care (CC) and public health (PH) services, which are financed by health insurance and direct government contributions, respectively. The achievement of strong PC is challenged by a shortage of PC providers. From 2009 to 2017, the number of PC providers in China increased from 1.83 to 2.51 million (1.37 to 1.80 PC providers per thousand population); the proportion of PC providers to the whole health system providers decreased from 36.43 to 30.22% during the same period, indicating the simultaneous reinforcement of secondary care [4, 5]. In addition, the breadth and volume of the workload of basic PH services expanded, causing an imbalance with the size of the PH workforce, thereby posing a threat to professionals’ health and quality of care [6]. The differences in roles and services delivery by clinical care and public health providers are presented in Additional file 1.

In the abovementioned context, an increasing amount of research has focused on solving retention problems and finding ways to improve the efficiency and productivity of providers, which may be affected by burnout of health workers. Health workers are at high risk of suffering from burnout, a risk that has only been increasing during the COVID-19 pandemic [7, 8]. Burnout among healthcare providers has been associated with adverse outcomes in patient care [9, 10], negative effects on the health worker’s health [11], reduced physician productivity, higher turnover intention and reduced patient access [12–16]. The most widely used definition of burnout is that from Maslach and Jackson. They defined burnout as a psychological syndrome of emotional exhaustion (i.e. feelings of being emotionally overextended and depleted of one’s emotional resource), depersonalisation (i.e. a negative, callous or excessively detached response to other people) and reduced personal accomplishment (i.e. a decline in one’s feelings of competence and successful achievement in one’s work), which can occur among individuals who work with other people [17].

Most of the previous studies on burnout among PC providers were conducted in other countries and across different type of providers (physicians, nurses, community health workers, midwives and pharmacists). The prevalence of burnout showed substantial variability across studies due to different measurements, target populations or contexts [18]. According to a systematic review published in 2018, the prevalence rates of emotional exhaustion, depersonalisation and decreased personal achievement were 27.4–99.6%, 13.3–98.0% and 25.1–99.3%, respectively, among PC providers in low- and middle-income countries [3]. Contributors to provider burnout are as follows: work-related factors, including excessive workload, inefficient work process, payment model, work support and autonomy; and individual factors, including gender, age and level of education [3, 16]. Incorporating a multilevel perspective into burnout research was argued to be more effective in confirming associated factors of burnout than focusing only at the individual level [19, 20].

Few studies have investigated burnout among PC providers in China [21–23]. Although these studies indicate an increased prevalence of burnout [21–23], they are limited as they mostly focus on a specific setting (e.g. township health centres representing rural areas or community health centres representing urban areas) [21, 22, 24, 25] or on regional (e.g. city or provincial level) contexts [19, 26, 27]; these studies pay little attention to different types of PC providers and define PC providers by their job category as opposed to their type of service provision. This approach does not do justice to the different types of services that are currently provided in the Chinese PC system and limits our understanding of factors that contribute to burnout. To our knowledge, not much is known about the prevalence of burnout among PC providers that are involved in CC or PH services in China. Thus, this study aims to measure the prevalence of job burnout among CC and PH service providers in China, to identify the associated factors of job burnout for the two types of PC providers and to determine the strategies to mitigate burnout.

## Methods

### Study design and sample

This cross-sectional study was performed in 2018 in six provincial regions in China. A multistage stratified cluster sampling was used to determine the sample of PC facilities. The detailed sampling strategy is published elsewhere [6]. All PC providers from the sample PC facilities (including township and community health centres) were invited to participate in the survey.

Data were collected via an online survey in collaboration with local health bureaus from October 2018 to November 2018. The local health bureaus helped the study team communicate with the PC facilities, and the director of each facility forwarded the online questionnaire to PC providers. Before answering the questionnaire, all participants were assured de-identification and confidentiality in handling their data and consent to participate. The organisational information was also collected from the director of each PC facility through an online survey. In total, 23,778 PC providers in 741 PC facilities participated in the survey. The response rate was 90.0% (23,778 out of 26,417). Cases with missing data in the surveys (209 cases in the provider survey and 753 cases on organisational information) or with wrong information (1057 cases with wrong facility name) were excluded. The final sample size was 21,759 PC providers from 708 PC facilities. The effective response rate was 82.4% (21,759 out of 26,417).

### Clinical care and public health service providers

In general, the daily work of PC providers includes service delivery and administrative work. Service delivery includes CC and PH services, and administrative work is only performed by some providers who hold an administrative position, e.g. (vice) director of the facility or head of a department in the facility. PC providers in China include doctors, nurses, pharmacists, clinical laboratory technicians and imaging technicians. Some of these providers undertake multiple positions or types of services. In the survey, participants were asked to estimate the average percentage of their working hours spent on CC service delivery, PH service delivery, traditional Chinese medicine service delivery and administrative work on a yearly basis. Given that traditional Chinese medicine services belong to CC, and the working hours of CC providers who only provide western medicine and those who only provide traditional Chinese medicine are the same at PC facilities, CC and traditional Chinese medicine service delivery were merged in this study. The participants were classified according to the estimated percentage of time they reportedly dedicate to three types of work, namely, CC service delivery, PH service delivery and administrative work. Those participants who dedicated more of their time to CC delivery than the other two types of work were included as CC providers (*n* = 13,512) in this study. Similarly, PH service providers (*n* = 4304) and administrative professionals (*n* = 1170) were determined and grouped. The average percentage of time spent in CC and PH service delivery in the CC and PH service groups were 77.7% and 78.2%, respectively. The distribution of the percentage for the groups is described in Additional file 2. The administrative group and participants who dedicated a large portion of their time equally to at least two of the three types of work (*n* = 973) and whose answers summed up larger than 100% (*n* = 1800) were excluded from this study. Overall, CC and PH service providers (*n* = 17,816) from 701 PC facilities were included in the analysis.

### Measures

#### Burnout

The Chinese version of the Maslach Burnout Inventory-General Survey (MBI-GS) was used, which was first introduced in 2002 [28] and has shown good reliability and validity in previous studies [21, 29, 30]. The 15-item scale measures three dimensions of job burnout, with 5 items for emotional exhaustion, 4 items for depersonalisation and 6 items for reduced personal accomplishment. Each item of the MBI-GS includes a 7-point Likert scale ranging from 0 (“never”) to 6 (“every day”). A high score for emotional exhaustion and depersonalisation and a low score for reduced personal accomplishment indicate more severe burnout. Therefore, the 6 items for reduced personal accomplishment were reversely scored. The following equation (1) was adopted to calculate the weighted sum score of burnout in consistency with other studies [29, 31, 32]:

1 $${\text{Burnout }} = \, \left( {0.{4}*{\text{ emotional exhaustion }} + \, 0.{3}*{\text{ depersonalisation }} + \, 0.{3}*{\text{ reduced personal accomplishment}}} \right)/{15}{\text{.}}$$

Cut-off points adopted by previous studies were used to classify the participants into the three following groups based on their score [31, 32], namely, group 1—no burnout (0–1.49), group 2—moderate burnout (1.50–3.49) and group 3—severe burnout (3.5–6.0). The criteria used to indicate the presence of emotional exhaustion, depersonalisation and reduced personal accomplishment were the average score of the items for subcomponents ≥ 3.2, > 2.2 and ≤ 4.0, respectively [33].

#### Associated factors of burnout

The associated factors included in this study can be divided into three categories, namely, demographics, occupational factors and organisational factors. The definitions and categories of the associated factors are shown in Table 1.

**Table 1:** Definitions and categories of the associated factors

Factors	Definitions and categories
*Demographics*	
Age*	Age of the participant
Gender	Gender of the participant 0 = male, 1 = female
Marital status	Marital status of the participant 0 = married, 1 = unmarried (including single, divorced or widowed)
Education level	Education level of the participant 0 = junior college or below, 1 = undergraduates, 2 = postgraduates
*Occupational factors*	
Job type	Job type of the participant 0 = doctors, 1 = nurses, 2 = pharmacist, 3 = others
Title	The rank of a professional post 0 = junior or no title, 1 = intermediate, 2 = senior
Administrative responsibility	If the participant has administrative responsibility or not 0 = yes, 1 = no
Employment status	The employment status of the participant 0 = permanent, 1 = temporary (including providers with temporary contracts and those rehired after retirement)
Working years*	Working years in current facility
Working hours	Working hours per week of the participant 0 = < 40, 1 = 40–49, 2 = 50–59, 3 ≥ 60
Income proportion	Proportion of the participant’s income to the total household income 0 = < 25%, 1 = 25–49%, 2 = 50–75%, 3 = ≥ 75%
*Organisational factors*	
Location	Location of the facility 0 = eastern, 1 = central, 2 = western
Facility type	Type of the facility 0 = township health centre (representing rural areas), 1 = community health centre (representing urban areas)
Performance-based salary*	The ratio of performance-based salary of the facility. The salary of PC facilities in China consisted of two parts, namely, the fixed and the fluctuating parts. The fluctuating part is performance-based salary

^*^Continuous variables

### Statistical analysis

The *t*-test and Chi-square test were used to compare the characteristics, burnout and its subcomponents scores between CC and PH service providers in Stata 15. Multilevel linear regression analysis was conducted to identify the associated factors of job burnout for CC and PH service providers in Stata 15, respectively. The dependent variables were the weighted sum score of burnout and the average score of each subcomponent. The null model (i.e. intercept-only model) was first generated for each dependent variable to test the existence of sufficient variance at the cluster level in influencing burnout. The interclass correlation coefficient was computed. Then, the full model, which was a two-level random intercept linear regression model with level one predictors (i.e. demographics and occupational factors) and level two predictors (i.e. organisational factors), was constructed. Goodness-of-fit of the models were examined by the log likelihood-ratio test. The multicollinearity between the predictors was evaluated by variance inflation factors, which demonstrated the existence of multicollinearity if larger than 10.

## Results

### Study participants and facilities

In total, 17,816 CC and PH service providers from 701 PC facilities were included in this study. Table 2 presents the description of participants and facilities. Among sample facilities, 45%, 21% and 34% were from eastern, central and western China, respectively, which were consistent with the national data (41%, 27% and 31%, respectively). The average age of the overall participants was 35 years old, and 70% of them were female. The results of Chi-square and t-test showed that the differences between CC and PH service providers for most individual characteristics (except for age, marital status and income proportion) were statistically significant (*p* < 0.05). Compared with the CC group, more women (75% vs 69%), more providers who graduated from junior college or below (61% vs 58%), fewer providers with junior or senior title (26% vs 31%), fewer providers with administrative responsibilities (30% vs 33%), less average working years (9.01 vs 9.15) and fewer providers working more than 50 h per week (30% vs 45%) were in the PH service group.

**Table 2:** Description of the participants (*n* = 17,816) and facilities (*n* = 701)

	Overall		CC		PH		*p*-value
	*N*	%	*N*	%	*N*	%	
*Individual*							
Age (mean, SD)	35.39	9.27	35.42	9.40	35.30	8.84	0.467^*^
Gender							
Male	5312	29.82	4239	31.37	1073	24.93	< 0.001^†^
Female	12,504	70.18	9273	68.63	3231	75.07	
Marital status							
Married	14,095	79.11	10,658	78.88	3437	79.86	0.169^†^
Unmarried	3721	20.89	2854	21.12	867	20.14	
Education level							
Junior college or below	10,479	58.82	7873	58.27	2606	60.55	0.016^†^
Undergraduates	7181	40.31	5525	40.89	1656	38.48	
Postgraduates	156	0.88	114	0.84	42	0.98	
Job type							
Doctor	8421	47.27	5767	42.68	2654	61.66	< 0.001^†^
Nurse	5343	29.99	4244	31.41	1099	25.53	
Pharmacist	1266	7.11	1117	8.27	149	3.46	
Others	2786	15.64	2384	17.64	402	9.34	
Title							
Primary	12,452	69.89	9266	68.58	3186	74.02	< 0.001^†^
Junior	4623	25.95	3619	26.78	1004	23.33	
Senior	741	4.16	627	4.64	114	2.65	
Administrative responsibility							
Yes	5780	32.44	4501	33.31	1279	29.72	< 0.001^†^
No	12,036	67.56	9011	66.69	3025	70.28	
Employment status							
Long term	12,920	72.52	9723	71.96	3197	74.28	0.003^†^
Temporary	4896	27.48	3789	28.04	1107	25.72	
Working years (mean, SD)	10.03	9.12	10.16	9.15	9.64	9.01	0.001^*^
Working hours							
< 40	1556	8.73	1156	8.56	400	9.29	< 0.001^†^
40–49	8867	49.77	6249	46.25	2618	60.83	
50–59	3892	21.85	3029	22.42	863	20.05	
≥ 60	3501	19.65	3078	22.78	423	9.83	
Income proportion							
< 25%	3365	18.89	2530	18.72	835	19.4	0.265^†^
25%–49%	5289	29.69	3981	29.46	1308	30.39	
50%–75%	5718	32.09	4354	32.22	1364	31.69	
> 75%	3444	19.33	2647	19.59	797	18.52	
*Facility*							
Location							
Eastern	315	44.94	–				
Central	148	21.11	–				
Western	238	33.95	–				
Facility type							
Township health centre	420	59.91	–				
Community health centre	281	40.09	–				
Performance-based salary (mean, SD)	37.28	18.68	–				

*p value of *t*-test to examine the significance of difference in the variable between clinical care and public health service groups

^†^*p* value of Chi-square test to examine the significance of difference in the variable between clinical care and public health service groups

### Prevalence of job burnout

Table 3 shows the prevalence of job burnout and the three subcomponents among CC and PH service providers. Overall, half of the providers (50.09%) suffered from burnout, and 2.99% of the providers had severe burnout. The presence of burnout and the proportion of severe burnout in the PH service group (58.06% and 5.25%) were higher than those in the CC group (47.55% and 2.26%, respectively). The prevalence of reduced personal accomplishment (40.85%) was larger than those of emotional exhaustion (14.60%) and depersonalisation (13.20%) among overall providers, and the prevalence of all three subcomponents among PH service providers was larger than among CC providers.

**Table 3:** Prevalence of job burnout and the subcomponents among clinical care (CC) and public health (PH) service providers

Degree	Overall (*N* = 17,816)		CC (*N* = 13,512)		PH (*N* = 4304)	
	*N*	%	*N*	%	*N*	%
*Job burnout*						
No burnout	8892	49.91	7087	52.45	1805	41.94
Moderate burnout	8392	47.10	6119	45.29	2273	52.81
Severe burnout	532	2.99	306	2.26	226	5.25
*Subcomponents*						
Emotional exhaustion	2602	14.60	1776	13.14	826	19.19
Depersonalisation	2351	13.20	1571	11.63	780	18.12
Reduced personal accomplishment	7278	40.85	5256	38.90	2022	46.98

Table 4 presents the average scores of burnout and its three subcomponents among CC and PH service providers. According to the results of *t*-tests, all scores (including weighted sum score of burnout and scores of the three subcomponents) of PH service providers were higher than the scores of CC providers.

**Table 4:** Burnout scores among clinical care (CC) and public health (PH) service providers

Degree	Overall (*N* = 17,816)		CC (*N* = 13,512)		PH (*N* = 4304)		*p* value^a^
	Mean	SD	Mean	SD	Mean	SD	
Weighted sum score of burnout	1.58	0.95	1.51	0.92	1.77	1.02	< 0.001
Emotional exhaustion	1.95	1.28	1.89	1.25	2.15	1.34	< 0.001
Depersonalisation	1.03	1.09	0.97	1.05	1.21	1.19	< 0.001
Reduced personal accomplishment^b^	4.38	1.36	4.44	1.36	4.19	1.38	< 0.001

^a^*p* value of t-test to examine the significance of difference in the variable between clinical care and public health service groups

^b^The scores of items of reduced personal accomplishment were not reversed in the analysis of single dimension

### Factors associated with job burnout

The intra-class correlation coefficient in the null model and the goodness-of-fit indicator for each dependent variable were presented in Additional file 3. For the 8 dependent variables, including the weighted sum score of burnout and the average score of each subcomponent for the CC and PH service groups separately, the intra-class correlation coefficients ranged from 0.07 to 0.2, which were all larger than 0.059. The tests of the preference of log likelihood versus linear regression were also strongly significant (*p* < 0.001), indicating that the multilevel linear regression was appropriate. The variance inflation factor for each predictor was smaller than 10, which demonstrated the absence of multicollinearity.

#### Demographics

Table 5 shows factors associated with burnout and its three subcomponents for CC and PH service providers. Age was negatively associated with burnout, emotional exhaustion, depersonalisation and reduced personal accomplishment in both groups (*p* < 0.05), except for emotional exhaustion in the PH service group (*p* > 0.05). Similarly, female providers showed a lower level of burnout and its subcomponents in both groups (*p* < 0.05), except for emotional exhaustion in the PH service group (*p* > 0.05). Marital status was not associated with burnout (*p* > 0.05), but unmarried providers indicated a lower degree of emotional exhaustion and personal accomplishment in the CC group (*p* < 0.05). Higher educational degree was related to a higher level of burnout, emotional exhaustion and depersonalisation in CC group (*p* < 0.05), while for PH service group, providers with the highest educational degree (postgraduates) showed no difference compared with providers with the lowest degree (junior college or below).

**Table 5:** Factors associated with burnout and its subcomponents for clinical care and public health service providers

	Clinical care				Public health services			
	Burnout	EE	DP	PA^a^	Burnout	EE	DP	PA^a^
Age	− 0.010**	− 0.005**	− 0.010**	0.018**	− 0.012**	− 0.002	− 0.009**	0.026**
Gender (ref: male)								
Female	− 0.074**	− 0.068**	− 0.066**	0.089**	− 0.100**	− 0.052	− 0.118**	0.142**
Marital (ref: married)								
Unmarried	0.002	− 0.092**	0.034	− 0.102**	0.031	− 0.027	0.069	− 0.084
Education (ref: junior college or below)								
Undergraduates	0.074**	0.173**	0.121**	0.099**	0.078**	0.145**	0.120**	0.041
Postgraduates	0.266**	0.262**	0.428**	− 0.114	0.217	0.186	0.219	− 0.286
Job type (ref: doctor)								
Nurse	− 0.074**	− 0.089**	− 0.054**	0.073**	− 0.143**	− 0.137**	− 0.141**	0.161**
Pharmacist	− 0.191**	− 0.279**	− 0.165**	0.100**	− 0.205**	− 0.145	− 0.171*	0.321**
Others	− 0.163**	− 0.178**	− 0.160**	0.147**	− 0.109**	− 0.173**	− 0.070	0.071
Title (ref: junior or no title)								
Intermediate	0.113**	0.204**	0.123**	0.011	0.202**	0.246**	0.248**	− 0.107*
Senior	0.072*	0.135**	0.091**	0.021	0.054	0.122	0.169	0.168
Administrative responsibility (ref: yes)								
No	0.073**	0.045**	0.103**	− 0.085**	0.032	− 0.055	0.088**	− 0.092**
Employment status (ref: permanent)								
Temporary	− 0.089**	− 0.129**	− 0.071**	0.042	− 0.104**	− 0.164**	− 0.121**	− 0.007
Working years	0.002*	0.002	0.003	− 0.002	0.001	0.002	− 0.003	− 0.003
Working hours (ref: < 40)								
40–49	0.091**	0.199**	0.088**	0.047	0.033	0.220**	0.026	0.203**
50–59	0.206**	0.418**	0.162**	0.029	0.161**	0.490**	0.105	0.212**
≥ 60	0.348**	0.775**	0.261**	0.133**	0.426**	0.954**	0.408**	0.254**
Income proportion (ref: < 25%)								
25–49%	− 0.105**	− 0.115**	− 0.113**	0.082**	− 0.138**	− 0.162**	− 0.187**	0.062
50–75%	− 0.034	0.037	− 0.058**	0.098**	− 0.047	0.001	− 0.072	0.074
> 75%	0.029	0.207**	0.002	0.172**	0.012	0.145**	− 0.018	0.120*
Location (ref: eastern)								
Central	− 0.145**	− 0.156**	− 0.141**	0.109**	− 0.218**	− 0.330**	− 0.260**	0.006
Western	0.036	− 0.084**	0.036	− 0.212**	0.090	0.016	− 0.056	− 0.354**
Facility type (ref: township health centre)								
Community health centre	0.155**	0.183**	0.135**	− 0.140**	0.154**	0.251**	0.127**	− 0.043
Performance-based salary	0.003**	0.004**	0.003**	− 0.001	0.003**	0.004**	0.003**	− 0.001
Constant	1.664**	1.553**	1.078**	3.651**	2.053**	1.828**	1.505**	3.146**

**p < 0.05, *p < 0.1

^a^The scores of items of reduced personal accomplishment were not reversed in the analysis of single dimension

#### Occupational factors

Doctors were related to a higher degree of burnout than other job types (*p* < 0.05) in both groups. Compared with providers with junior or no title, providers with intermediate title in both groups showed a higher level of burnout (*p* < 0.05), whereas senior providers showed no difference in both groups (*p* > 0.05). Providers in both groups with temporary contract showed a lower level of burnout (*p* < 0.05) compared with permanent providers, as well as lower emotional exhaustion and depersonalisation (*p* < 0.05). Working hours were positively associated with the level of burnout, emotional exhaustion and depersonalisation for CC providers (*p* < 0.05) and with the level of burnout, emotional exhaustion and personal accomplishment for PH service providers (*p* < 0.05).

#### Organisational factors

For facility-level factors, community health centres predicted a higher level of burnout, emotional exhaustion, depersonalisation and reduced personal accomplishment in both groups (*p* < 0.05), except for reduced personal accomplishment in the PH service group (*p* > 0.05). Performance-based salary was positively associated with burnout, emotional exhaustion and depersonalisation in both groups (*p* < 0.05). Comparing with PC facilities from eastern China, those from central China showed a lower level of burnout (*p* < 0.05), whereas those from western China showed no difference (*p* > 0.05).

## Discussion

This study aimed to measure the prevalence of job burnout and identify its associated factors among CC and PH service providers at PC facilities in China. Half of the providers suffered from burnout, and more PH service providers showed moderate or severe burnout than CC providers. Similar factors were related to job burnout between the two provider groups, including demographics (age, gender and education level), occupational (job type, professional title and working hours) and organisational factors (location, facility type and performance-based salary).

This study showed that the self-reported prevalence of burnout among PC providers in 2018 was 50.09%, indicating that burnout is prevalent in PC facilities in China. This result is consistent with those of previous small-scale studies on job burnout among Chinese PC providers, as measured by MBI-GS, although the prevalence rate of burnout in our study was higher than those in previous studies (27.8 to 39.7%) conducted between 2010 and 2015 [23, 34–36]. This may indicate an increasing trend of burnout among PC providers in China. Despite the policy focus on strengthening PC, we have observed a decreasing proportion of PC workforce to the overall health system workforce in China; moreover, shortage of workforce has been reported [37–39], whereas task profiles of PC staff have been largely expanded under recent reforms. This increase in experienced workload of PC providers may lead to burnout. The prevalence of moderate burnout in this study (47.1%) is much lower than that (70.25%) shown by a recent published study from Hubei Province, China [26], which may be due to the study scale (national vs. regional) or the different instruments adopted (MBI-GS vs. MBI-HSS) to measure burnout. The results of this current study demonstrated that the prevalence of reduced personal accomplishment was higher than that of the other two subcomponents, which is in line with the results of previous studies conducted at Chinese PC facilities [19, 26, 33]. Reported low levels of personal accomplishment among Chinese PC providers might be explained by the fact that citizens are free to choose their first-contact health care facility; they generally have little trust in PC and usually bypass PC facilities to seek health care in hospitals [19]. Specifically, the prevalence rates of emotional exhaustion (14.60%) and depersonalisation (13.20%) in this study were lower than their counterparts in low- and middle-income countries (27.4–99.6% and 13.3–98.0%, respectively), whereas those of reduced personal accomplishment (40.85%) was within the range of 25.1–99.3% [3].

The reported prevalence rates of burnout and its subcomponents among PH service providers were higher than those of CC providers. One possible explanation is that PH service providers accept more workload and spend more time on administrative tasks (e.g. filling up health records and follow-up forms), which are not the most rewarding tasks [40, 41]. Our previous study showed that the volume-based output of PH service providers was increasingly larger than that of CC service providers in 2009–2015, but the gap became slightly smaller after 2015; the output of PH service providers still remains 4.6 times that of CC service providers [6]. Thus far, CC and PH services at PC facilities in China are relatively fragmented in terms of service delivery (i.e. providers in charge of different services seldom communicate with one another [42]) and financing system (i.e. health insurance schemes cover basic CC, whereas government subsidies finance basic PH services), which may hinder the distributive justice of work between the two service groups and the collaborative and friendly working environment. Studies have confirmed the impact of distributive justice and working environment on job burnout [3, 16, 28], which will ultimately affect the improvement in health outcomes for patients—the core objective of the primary health care system. Therefore, the PH service providers need to receive more attention, and the synergy between CC and PH services needs to be strengthened by increasing flexibility in care substitution, enhancing team-based work and integrating the financing systems.

Overall, similar factors seemed to be associated with job burnout between CC and PH service groups in this study, with only a few exceptions. In accordance with previous studies, age was negatively related to burnout, and this can be explained by the classical Job Demands–Resources model. The lack of resources to cope with demanding situations at work was associated with depersonalisation and reduced personal efficacy [43]. In general, younger health care workers have less work experience, network support and work autonomy [44, 45], which may result in job burnout and the appearance of symptoms of burnout subcomponents among early age PC providers. Male providers reported a higher level of burnout, which is in line with the findings of a recent study that focused on Chinese PC providers [19]. With higher expectations and stress, providers with higher education background were more likely to suffer from burnout [45]. Different from the CC group, the burnout scores of postgraduates from PH service group were not significantly distinct from the reference (i.e. junior college or below), which may be due to the smaller sample size (*n* = 42).

Doctors, providers with intermediate professional title and providers with a permanent contract and more working hours, reported a higher level of burnout. Interestingly, providers with intermediate professional titles were more likely to show burnout than the reference (i.e. junior or no title), but the senior providers showed no difference from the reference. Although the senior providers presented a higher degree of emotional exhaustion and depersonalisation, the coefficients (0.135 and 0.091) were still smaller than those of the intermediate group (0.204 and 0.123, respectively). One explanation may be that providers with an intermediate title are generally in the promotion period of their career when their ambitions, competition stress and even unmet expectations may all lead to burnout. As expected, the longer their working hours per week, the higher the level of burnout of the PC providers. More than 90% of providers in both CC and PH service groups worked more than 40 h per week, and 22.78% of CC and 9.83% of PH service providers worked more than 60 h per week, indicating that the shortage of workforce and the inefficiency of care delivery in the PC system need to be addressed. Another interesting result of this study was that more working hours were associated with high personal accomplishment, which needs to be further confirmed, as some studies suggested that the dimension of reduced personal accomplishment is not as important as the other two dimensions in explaining job burnout and may not be a part of the concept of burnout [46, 47]. The present study found that PC providers with a temporary contract reported a lower degree of burnout, which was inconsistent with the findings of other studies [19, 26, 44]; this inconsistency may be due to the fact that the temporary contract group in this study included providers who have been rehired after retirement.

All three facility factors (including region, facility type and performance-based salary) were associated with burnout. Community health centres were more likely to have a higher level of burnout than township health centres. Related conclusions from previous studies were mixed [23, 26, 48]. Though some studies showed opposite results, the rapid urbanisation in China is challenging the health care system in urban cities because of the increasing population, which may add to workload of health care providers. Moreover, the higher level of job demand and the lower level of job control in large cities may also lead to burnout compared with small cities, towns or villages [48]. Performance-based salary was positively related to overall burnout, emotional exhaustion and depersonalisation. This finding may be due to the following two facts. Firstly, the government is using more process-oriented and volume-based indicators (e.g. number of outpatient visits and filling follow-up health records) rather than outcome-oriented ones to evaluate the performance of providers. The performance-based salary system was established in 2009, and it may lead to a higher degree of emotional exhaustion among PC providers [42, 49]. Secondly, the performance evaluation system focuses more on individual incentives rather than team-based ones, consequently increasing competition between colleagues and impacting peer relationships. This system may further lead to depersonalisation among PC providers. Therefore, we recommend that the performance-based salary system be transformed into a quality-based one, and teamwork incentives are needed to encourage collaboration [50].

This study has strength and limitations. A strong point of this study is that large samples of PC providers from six provinces have been surveyed. Moreover, to our knowledge, this is the first study to compare the job burnout between CC and PH service providers in China. With the multilevel linear regression model, we considered a nested data structure. However, we asked the participants to estimate the percentage of their participation in four types of services on a yearly basis, which may not result in a precise answer due to recall bias. However, this approach is more practical and understandable for PC providers in China, because job substitution is not a daily or monthly routine for many PC providers. For example, CC providers may participate in some health education activities that could be organised randomly within a year by PC facilities. Furthermore, although MBI-based instruments are the most widely used among a variety of measurements for job burnout, many different definitions of overall burnout prevalence and the prevalence of its subcomponents exist [3, 18]. Thus, comparison between this study’s results and those of other studies conducted in low- and middle-income countries must be interpreted with caution.

## Conclusions

Job burnout is prevalent among different types of PC providers in China, thereby requiring action toward PC in general. More PH service providers are experiencing burnout than CC providers. Similar factors were associated with burnout among CC and PH service groups. We recommend strengthening the synergy between CC and PH services by increasing flexibility in care substitution, encouraging team work and integrating the financing systems. The organisational environment, which includes the incentive policy of the performance-based salary, affects job burnout in addition to workload and job type. We recommend that the performance-based salary system be transformed into a quality-based system that includes teamwork incentives. Younger, male and lower-educated PC providers and providers with an intermediate professional title or permanent contract were the high-risk groups. Further research is required to understand why burnout is higher among these specific groups and to ensure that effective support and coping strategies are provided.

## Supplementary information

**Additional file 1.** Roles of and services provided by clinical care and public health services providers. Additional file 1 presents a table showing the roles of and services provided by clinical care and public health services providers.

**Additional file 2.** Distribution of the percentage for clinical care, public health service providers and administrative group. Additional file 2 presents three tables showing the distribution of the percentage for clinical care (*n* = 13512), public health service providers (*n* = 4304) and administrative group (*n* = 1170), respectively.

**Additional file 3.** Parameter coefficients and test of goodness-of-fit of multilevel models (null models). Additional file 3 presents a table showing the intra-class correlation coefficient in the null model and the goodness-of-fit indicator for each dependent variable.

## Data Availability

The datasets used and/or analysed during the current study are available from the corresponding author on reasonable request.

## Abbreviations

PC: Primary care
CC: Clinical care
PH: Public health
MBI-GS: Maslach Burnout Inventory-General Survey
